# Type-1 (CB_1_) Cannabinoid Receptor Promotes Neuronal Differentiation and Maturation of Neural Stem Cells

**DOI:** 10.1371/journal.pone.0054271

**Published:** 2013-01-23

**Authors:** Claudia Compagnucci, Sara Di Siena, Maria Blaire Bustamante, Daniele Di Giacomo, Monia Di Tommaso, Mauro Maccarrone, Paola Grimaldi, Claudio Sette

**Affiliations:** 1 Department of Biomedicine and Prevention, Section of Anatomy, University of Rome “Tor Vergata”, Rome, Italy; 2 Laboratory of Neuroembryology, Fondazione Santa Lucia IRCCS, Rome, Italy; 3 Laboratory of Lipid Neurochemistry, Fondazione Santa Lucia IRCCS, Rome, Italy; 4 Department of Biomedical Sciences, University of Teramo, Teramo, Italy; Universitätsklinikum Carl Gustav Carus an der Technischen Universität Dresden, Germany

## Abstract

Neural stem cells (NSCs) are self-renewing cells that can differentiate into multiple neural lineages and repopulate regions of the brain after injury. We have investigated the role of endocannabinoids (eCBs), endogenous cues that modulate neuronal functions including neurogenesis, and their receptors CB_1_ and CB_2_ in mouse NSCs. Real-time PCR and Western blot analyses indicated that CB_1_ is present at higher levels than CB_2_ in NSCs. The eCB anandamide (AEA) or the CB_1_-specific agonist ACEA enhanced NSC differentiation into neurons, but not astrocytes and oligodendrocytes, whereas the CB_2_-specific agonist JWH133 was ineffective. Conversely, the effect of AEA was inhibited by CB_1_, but not CB_2_, antagonist, corroborating the specificity of the response. CB_1_ activation also enhanced maturation of neurons, as indicated by morphometric analysis of neurites. CB_1_ stimulation caused long-term inhibition of the ERK1/2 pathway. Consistently, pharmacological inhibition of the ERK1/2 pathway recapitulated the effects exerted by CB_1_ activation on neuronal differentiation and maturation. Lastly, gene array profiling showed that CB_1_ activation augmented the expression of genes involved in neuronal differentiation while decreasing that of stemness genes. These results highlight the role of CB_1_ in the regulation of NSC fate and suggest that its activation may represent a pro-neuronal differentiation signal.

## Introduction

In the developing brain, precursor cells -neuroprogenitors or neural stem cells (NSCs)- yield functional neurons and glial cells. This process, named neurogenesis, mainly occurs during embryo development and perinatally, even though the ability to generate new neurons is maintained throughout adulthood in restricted areas of the nervous system [1]. For instance, the subventricular zone (SVZ) of the lateral ventricles and the subgranular zone (SGZ) in the dentate gyrus of the hippocampus contain NSCs. These stem cells can differentiate into neurons and glial cells in response to specific cues [2], [3]. Importantly, the newborn neurons have the potential to migrate in different areas of the brain where they can functionally integrate into the pre-existing neuroanatomical circuitries [1], [4]. This feature of adult neurogenesis has attracted considerable attention for the possible application of NSCs in the treatment of neurodegenerative diseases or brain injuries.

NSCs are physiologically subjected to different signals coming from the *in vivo* environment that are fundamental for their cell fate. Several morphogens produced in the niche hosting the NSCs can induce their proliferation, thereby maintaining their number in the brain, or can trigger their differentiation in various neural lineages [5]. In this regard, a key role for the endocannabinoid (eCB) system in the regulation of neurogenesis [6], neuronal activity [7] and NSC function [8] has recently emerged. The two main eCBs are *N*-arachidonoylethanolamine (anandamide, AEA) and 2-arachidonoylglycerol (2AG), which exert most of their biological activities by binding to type-1 (CB_1_) and type-2 (CB_2_) cannabinoid receptors [9]. The CB_1_ receptor is widely expressed in the brain, with higher levels detected in the hippocampus, the cerebellum and in the basal ganglia [10], [11], whereas expression of the CB_2_ receptor is restricted in the brain and more abundant in the immune system [12]–[14]. Cannabinoid receptors are coupled to G_i_/G_o_-proteins and their activation leads to inhibition of adenylyl cyclase and, in the case of CB_1_, to modulation of ion channels, including inhibition of voltage-dependent Ca^2+^ channels and activation of inwardly rectifying K^+^ channels [9], [15]. CB receptors also trigger signaling pathways involved in the regulation of cell fate, such as the mitogen-activated protein kinase (MAPK) family (ERK, JNK and p38) and the phosphoinositide-3 kinase (PI3K)/AKT pathways [9], [15], [16]. In addition, activation of CB_1_ or CB_2_ receptor in the neural context has been recently shown to modulate the mammalian target of rapamycin (mTOR) signaling both *in vivo* and *in vitro* [17], [18]. eCBs are produced on demand by activated postsynaptic cells and exert a modulatory effect by controlling neurotransmitter release via presynaptic activation of the CB_1_ receptor [7], [19]. Moreover, eCBs were shown to protect neurons from different brain insults such as ischemia, cerebral trauma and oxidative stress [20], and to regulate neural cell survival [21].

A role for the eCB system, and in particular for CB_1_-activated signalling, in the control of neurogenesis has been recently proposed. Neurogenesis involves both proliferation of progenitor cells and their differentiation into new neurons. Treatment of embryonic hippocampal neural progenitor cells with the synthetic agonist HU210 promoted proliferation without affecting differentiation [22]. Moreover, chronic treatment in vivo with the same agonist increased neurogenesis in the hippocampal dentate gyrus of adult rats and induced anxiolytic and antidepressant-like effects [22]. Another synthetic cannabinoid agonist, WIN-55,212-2, was also shown to promote embryonic neural progenitor cell proliferation and the generation of neurospheres in culture, and this effect was abolished in CB_1_- deficient progenitor cells [23]. Lower levels of neural progenitor proliferation were also observed in vivo in adult CB_1_-deficient mice [24] and in wild type mice treated with the CB1 antagonist SR141716 [25], suggesting that the role played by CB_1_ was maintained postnatally. On the other hand, the effect of eCBs on neural cell differentiation is less clear. In one study, prolonged treatment with AEA of neuronal progenitor cells (isolated from 7 days post partum mice) appeared to induce neuronal differentiation [26]. However, another study showed that AEA inhibited in vitro differentiation of embryonic neural progenitor cells through the CB1 receptor and the effect was mediated by attenuation of the extracellular signal regulated kinase pathway [27]. They also showed that the AEA analogue methanandamide significantly decreased adult neurogenesis in vivo, whereas the opposite effect was observed by blocking the CB_1_ receptor with SR141716 [27]. It is interesting to note that both the knockout of CB_1_ receptor [24] and its chronic stimulation by methanandamide [27] negatively affected neurogenesis, suggesting that this process requires a fine balance of CB_1_ receptor activation.

In this study, we have investigated the possible role of the eCBs on NSC differentiation. We found that agonists of the CB_1_ receptor enhance NSC differentiation and maturation into neurons. CB_1_ agonist-induced neuronal differentiation is coupled to a long-term decrease in the level of phosphorylation of ERK1/2 and treatment of NSCs with an inhibitor of this signalling pathway recapitulates the effect exerted by CB_1_ agonists on neuronal differentiation. Importantly, CB_1_ activation enhanced the expression of a subset of genes involved in neuronal differentiation. These findings uncover a potential role for eCBs in the modulation of NSC differentiation.

## Materials and Methods

### Animals and neural stem cells isolation and culture

Neural stem cells (NSCs) were isolated from C57/BL6 (Charles River Laboratories, Sulzfeld, Germany) mouse embryos, following the Institutional guidelines of the University of Rome Tor Vergata and the approval of the Ethical Committe. The age of the embryos was determined according to the staging criteria of Theiler, in which embryonic day 13.5 (E13.5) corresponds to stage 22 [28]. E13.5 embryos were removed from the maternal uterus and brains were dissected and transferred to minimal essential medium (MEM; Sigma-Aldrich, St. Louis, MO, USA). Meninges and olfactory bulbs were removed, and cerebral cortices were isolated. Tissues were treated as reported [29], [30]; in particular, they were enzymatically digested with Papain (30 U/ml, Worthington, Lakewood, NJ, USA), L-Cysteine (0.24 mg/ml, Sigma-Aldrich, St Louis, MO, USA), DNaseI (40 µg/ml, Sigma-Aldrich, St Louis, MO, USA) dissolved in MEM for 10 min at 37°C to obtain E13.5 embryonic cell suspensions. The enzyme activity was stopped by the addition of 1 ml of ovomucoid, containing 1 mg/ml Trypsin inhibitor (Sigma-Aldrich, St. Louis, MO, USA), 50 µg/ml bovine serum albumin (BSA), 40 µg/ml DNaseI in L-15 medium (all reagents from Sigma-Aldrich, St Louis, MO, USA). Cells were then centrifuged and resuspended in neurosphere medium consisting of DMEM∶F12 (1∶1) (Sigma-Aldrich, St. Louis, MO, USA) containing 0.2 mg/ml L-glutamine (Sigma-Aldrich St Louis, MO, USA), B27 (1 ml/50 ml, Gibco, Life Technologies Ltd, Paisley, UK), penicillin (100 U/ml), streptomycin (100 µg/ml) (all from Lonza, Basel, CH), and supplemented with epidermal growth factor (EGF) and basic fibroblast growth factor (bFGF) (both from EMD Millipore Corporation, Billerica, MA, USA). For the differentiation studies, four-well dishes (Greiner, Kremsmunster, Austria) were coated with poly-ornithine (Sigma-Aldrich, St Louis, MO, USA) in H_2_O, and with laminin-1 (Tebu-bio, Offenbach, Germany) in PBS for 1 h each at 37°C [30]. After the dishes were washed, the cells were plated at a density of 20000 cells/well in neurosphere medium containing 1% v/v fetal bovine serum (FBS) and incubated in a humidified atmosphere with 6% CO_2_ at 37°C for 3 days.

Anandamide (*N*-arachidonoylethanolamine, AEA) was purchased from Sigma-Aldrich (St Louis, MO, USA), arachidonoyl-2′-chloroethylamide (ACEA), 6-iodo-2-methyl-1-[2-(4-morpholinyl)ethyl]-1H-indol-3-yl](4-methoxyphenyl)methanone (AM630) and 1-(2,4-dichlorophenyl)-5-(4-iodophenyl)-4-methyl-N-1-piperidinyl-1H-pyrazole-3-carboxamide (AM251) were from Cayman Chemical (Ann Arbor, MI, USA), (6aR,10aR)-3-(1,1-Dimethylbutyl)-6a,7,10,10a-tetrahydro-6,6,9-trimethyl-6H-dibenzo[b,d]pyran (JWH133) was from Tocris-Cookson (Bristol, UK); U0126 was purchased from Calbiochem (Darmstadt, Germany). NSCs were treated with each drug as described in the text and in the Figure Legends of the corresponding experiments.

### Immunocytochemistry of dissociated neural stem cells

Immunofluorescence analyses were performed at room temperature as previously described [30]. The primary antibodies were incubated for 30 min at the following concentration: mouse anti-Nestin (1∶1000) (Millipore, Darmstadt, Germany), mouse anti-β-III-tubulin (1∶300) (Sigma-Aldrich, St Louis, MO, USA), mouse anti-O4 (1∶50) (generously provided by Alexander von Holst), and rabbit anti- GFAP (glial fibrillary acidic protein) (1∶250) (DAKO, Glostrup, Denmark). Cy3- (1∶500) and FITC-conjugated (1∶250) secondary antibodies (Jackson ImmunoResearch Laboratories, Inc. West Grove, PA, USA) were incubated for 1 hour. Samples were then viewed and photographed by using an inverted microscope (DMI6000B; Leica Geosystems AG, Heerbrugg, CH) equipped with a Pan-Neofluar 20x/0.75 objective lens. For TUNEL analyses, NSCs plated on coated dishes [30] were cultured for three days in medium containing 1% FBS and then analysed for DNA fragmentation with the terminal deoxynucleotidyl transferase (**T**dT)-mediated d**U**TP– fluorescein **n**ick **e**nd-**l**abelling (TUNEL, In Situ Cell Death Detection Kit, Fluorescein, Roche, Basel, CH) according to manufacturer's instructions. The cells were rinsed, mounted with cover-slip and analysed as described above.

### Morphometric measurements on neurites

Neurite length was estimated using a LAS AF software and the manual tracing feature was calibrated to display dimensions in microns on β-III tubulin-stained neurites. Branching level was determined by counting the number of primary, secondary, and tertiary neurites for each cell. Only neurons whose soma and processes were completely included in the captured images were analysed. Primary neurites were defined as processes projecting directly from the cell body and this has been considered as branching level 0, secondary neurites were processes that branched from any primary neurite (branching level 1) and tertiary processes were those that projected from any secondary neurite (branching level 2) (Fig. S2A).

### SDS–PAGE and Western blot analyses

Preparation of cell extracts and Western blot analyses were performed as previously described [31] using the following primary antibody: mouse anti-β-tubulin (1∶1000, Sigma-Aldrich, St Louis, MO, USA); rabbit anti-rpS6 and anti-p-ERK1/2 (1∶1000, Cell Signaling Technology, Darmstadt, Germany); rabbit anti-p-rpS6, anti-p-p38, and anti-pAKT (1∶1000, Biosource, Life Technologies Ltd, Paisley, UK). Signals were detected by enhanced chemiluminescence (ECL; SantaCruz Biotechnology, Santa Cruz, CA, USA).

### NSCs gene expression profile after ACEA-treatment

NSCs were cultured *in vitro* in differentiating condition (with 1% FBS) in the presence or absence of 1 µM ACEA for 24 hours. Total RNA was isolated from NSCs using the RNeasy mini kit (QIAGEN, Hilden, Germany) and transcribed to cDNA using the RT^2^ first-stand synthesis kit (SABiosciences, QIAGEN, Hilden, Germany). cDNA was used to perform PCR array analysis according to manufacturer's protocol with RT^2^ Real-Time SYBR Green PCR Master Mix using the ABI 7300 sequence detector system (Applied Biosystem, Foster City, CA). Expression of 84 genes was analyzed using the Neurogenesis and Neural Stem Cell PCR array (PAMM-404, SABiosceinces). Data were normalized using multiple housekeeping genes and analyzed by comparing 2^−ΔCt^ of the normalized data. Fold changes were calculated relative to control non-treated NSCs. The results were confirmed by quantitative real-time PCR (qPCR) analysis of seven genes.

### RT–PCR and real time PCR analysis

Total RNA (0.2–1 µg) from NSCs was used for RT–PCR using M–MLV reverse transcriptase (Invitrogen, Invitrogen, Life Technologies Ltd, Paisley, UK). Five percent of the reaction was used as template together with the primers specific for *CB_1_*, *CB_2_*, *Drd2*, *Slit2*, *Gdnf*, *Pax5*, *Shh*, *Dll1*, *Nestin*, *Pax6*, *Sox2*, *Gapdh*, and *β-actin* (Table 1). qPCR analysis was performed using SYBR Green I Master and the LightCycler 480 System (Roche, Basel, CH) or the ABI PRISM 7700 sequence detector system (Applied Biosystem, Foster City, CA), according to manufacturer's instructions. The ΔΔCt method was used to calculate the fold change in gene expression. ΔCt values were obtained by subtracting the Ct value obtained for the specific gene from the Ct value of the housekeeping gene for each sample and normalized to levels of the housekeeping genes (*Gapdh* and *β-actin*). Data represent fold increase *versus* control sample calculated by the 2_t_^−(ΔΔC)^ formula.

**Table 1:** List of the primers used for qPCR analyses.

Gene	PCR primers
CB_1_-R	Fw: 5′-CCAAGAAAAGATGACGGCAG-3′Rev: 5′-AGGATGACACATAGCACCAG-3′
CB_2_-R	Fw: 5′-TCGCTTACATCCTTCAGACAG-3′Rev: 5′-TCTTCCCTCCCAACTCCTTC-3′
Drd2	Fw: 5′-GTGTGCCATCAGCATCGACAG-3′Rev: 5′-CAGGACCCAGACAATGGCG-3′
Pax5	Fw: 5′-GTTGGCAGAGCGAGTCTGTG-3′Rev: 5′-GACACCTGCGTCACGGAG-3′
Slit2	Fw: 5′-GCACTGCCCCGCTGCTTGTA-3′Rev: 5′-AGGCTCCTGGAGGGATGACCC-3′
Gdnf	Fw: 5′-GGTGGCTACCTTCCTCCTTC-3′Rev: 3′-TTGCATTCCTGCTACAGTGC-3′
Shh	Fw: 5′-GGCTCGCCTGGCTGTGGAAG-3′Rev: 5′-ACGCGGTGTCCGGGACGTAA-3′
Dll1	Fw: 5′-ACATGTTCCTGCCGACCTGGGTA-3′Rev: 5′-CATGGCGCTCAGCTGACAGACCT-3′
Pax6	Fw: 5′-GTCACAGCGGAGTGAATCAGC-3′Rev: 5′-CGGGAAATGTCGCACGGC-3′
Sox2	Fw: 5′-CACAACTCGGAGATCAGCAA-3′Rev: 5′-CTCCGGGAAGCGTGTACTTA-3′
Nestin	Fw: 5′-GGTGGGCAGCAACTGGC-3′Rev: 5′-GGCTTTCCTGACCCCAAGCTG-3′
β-Actin	Fw: 5′-CTGTCGAGTCGCGTCCACCC-3′Rev: 5′-GCTTTGCACATGCCGGAGCC-3′
Gapdh	Fw: 5′-GTGGCAAAGTGGAGATTGTTGCC-3′Rev: 5′-GATGATGACCCTTTTGGCTCC-3′

## Results

### Expression of cannabinoid receptors in embryonic cortical neural stem cells

**Figure 1:**
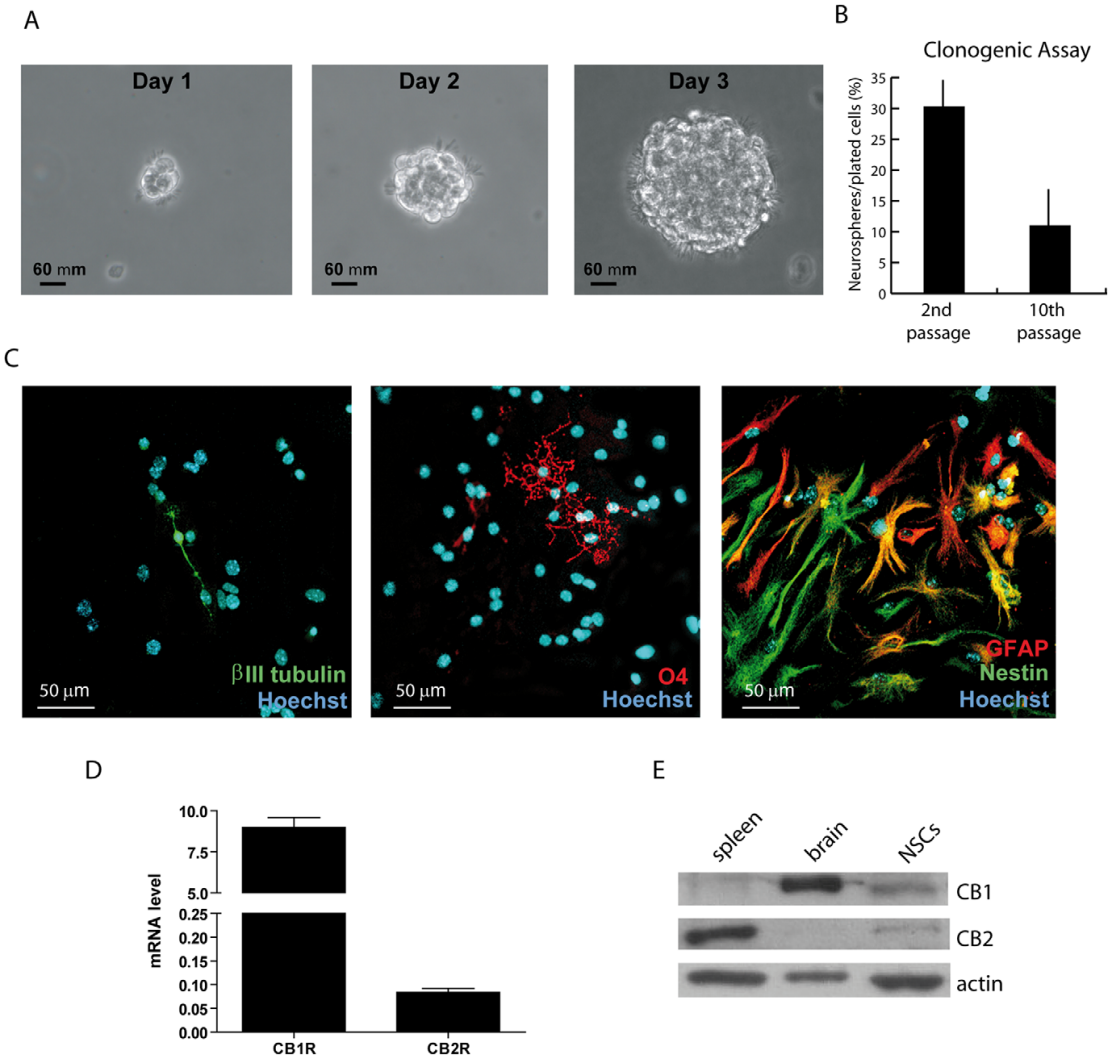
Neural stem cells (NSCs) express high levels of CB_1_ receptor. (**A**) Bright field photographs of murine NSCs forming neurospheres from single cell suspensions after one, two and three days in EGF and b-FGF. (**B**) Bar graph representing the clonogenic capacity of NSCs at the 2^nd^ and 10^th^ passage (n = 6). (**C**) Immunofluorescence analysis showing NSCs cultured in differentiating conditions (devoid of EGF and b-FGF, and supplemented with 1% FCS) and stained with the neuronal marker β-III tubulin (left panel), the oligodendrocyte marker O4 (middle panel), and co-stained with the astrocyte marker GFAP (red in the right panel) and with the neural progenitor marker Nestin (green in the right panel). Cells positive for both GFAP and Nestin were considered neural progenitors that are differentiating toward astrocytes (*i.e.* immature astrocytes). (**D**) qPCR analysis showing the levels of CB_1_ and CB_2_ receptor transcripts, normalized for levels of the housekeeping gene β-*actin*. (**E**) Western blot analysis showing the expression levels of CB_1_ and CB_2_ receptor proteins in extracts obtained from NSCs, brain and spleen.

The levels of expression of cannabinoids receptors CB_1_ and CB_2_ were investigated in a population of self-renewing cells isolated from the cortex of 13.5 murine embryos (E13.5). In the presence of minimum medium supplemented with epidermal growth factor (EGF) and basic fibroblast growth factor (bFGF), these cells maintain the ability to grow clonally and form floating spheres (Fig. 1A). At early passages (2–5), up to 30% of the plated cells yielded neurospheres, whereas with increasing passages their clonogenic potential slowly declined (Fig. 1B). Furthermore, upon depletion of growth factors and addition of 1% fetal bovine serum (FBS) [30], early passage cells plated onto laminin/poly-ornithine substrate ceased to proliferate and underwent differentiation into neurons (β-III tubulin-positive cells), oligodendrocytes (O4-positive cells) and astrocytes (GFAP-positive cells) (Fig. 1C). Thus, these cells display properties of NSCs [30],[32] and were used for further studies.

Quantitative real time PCR (qPCR) analysis indicated that the CB_1_ transcript is expressed at much higher levels in mouse NSCs than that of CB_2_ (Fig. 1D). To determine the corresponding protein expression levels, we performed Western blot analyses using adult brain extracts as positive control for the expression of CB_1_ receptor and spleen extracts as positive control for CB_2_ receptor. We found that CB_1_ was readily detectable in NSC extracts, whereas a fainter signal was detected for CB_2_ in NSCs isolated from the mouse embryonic cortex (Fig. 1E).

### Activation of CB_1_ receptor promotes neuronal differentiation

**Figure 2:**
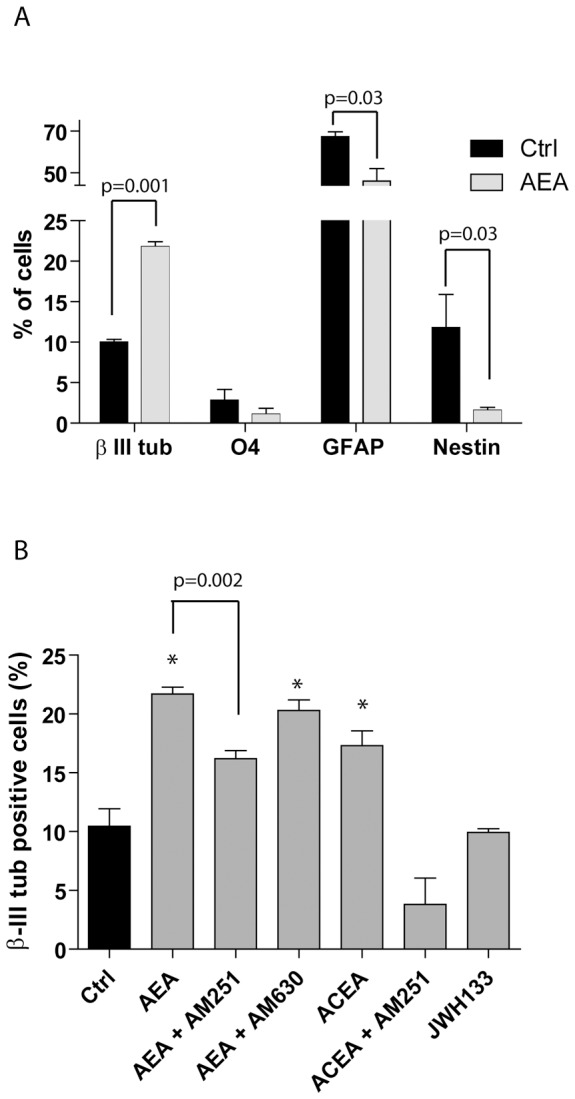
CB_1_ receptor activation promotes neuronal differentiation of NSCs *in vitro*. (**A**) The bar graph represents quantitative data (expressed in % of cells) of the immunofluorescence analysis of the effect exerted by anandamide (AEA) on NSCs cultured in differentiating condition. Immunofluorescence analysis was performed as described in Figure 1C to detect neurons (β-III positive cells), oligodendrocytes (O4-positive cells), astrocytes (GFAP-positive cells) and progenitor cells (Nestin-positive cells) in control (black bars) and AEA-treated (grey bars) cells. Data represents the mean ± standard deviation (SD) of 4 experiments. Statistical analysis was performed using the paired t-test; p value of statistically significant differences is reported above the bars. (**B**) The bar graph represents quantitative data (expressed in % of β-III tubulin-positive cells) of the immunofluorescence of neuronal differentiation of NSCs cultured in differentiating condition for 3 days in the presence or absence of AEA (1 µM), with or without the CB_1_ (AM251, 1 µM) or CB_2_ (AM630, 1 µM) antagonist, ACEA (1 µM) with or without the CB_1_ antagonist (AM251, 1 µM), and JWH133 (1 µM). Data represent the mean ± SD of 4 experiments. Statistical analysis of control *versus* treatment was performed using the paired t-test; * = p≤0.05, the p value of direct comparison of AEA+AM251 is indicated above the bars grouped by brackets. Where not indicated, samples did not show statistically significant changes.

We set out to investigate the possible involvement of AEA, an endogenous ligand that binds with similar affinity to CB_1_ and CB_2_ [9], [15], in NSC differentiation. After three days of culture, NSC differentiation was evaluated by immunofluorescence analysis using specific markers for neurons, astrocytes and oligodendrocytes (Fig. 1C). When NSCs were grown in minimal medium supplemented with EGF and bFGF (proliferation conditions, see Materials and Methods), more than 50% of the cells retained the undifferentiated state and expressed the precursor marker Nestin (Fig. S1A). Under these conditions, some cells began to express the neuronal marker β-III tubulin and the astrocyte marker GFAP. Notably, AEA (1 µM) significantly increased the percentage of β-III tubulin-positive cells (4.76±0.90 in AEA-treated cells vs 2.26±0.50 in control cells), suggesting that it promotes neuronal differentiation. To directly test the effect of AEA on differentiation, NSCs were plated onto laminin/poly-ornithine and cultured in the presence of 1% FBS to block proliferation and promote differentiation [30]. Under these conditions, AEA significantly promoted NSC differentiation into neurons (21.79±1.12 in AEA-treated cells vs 10.00±0.61 in the control cells) (Fig. 2A). Treatment with AEA also mildly decreased the percentage of GFAP-positive astrocytes (46.00±10.51 in AEA-treated cells vs 64.34±4.15 in control cells), whereas it strongly decreased the percentage of cells that remained undifferentiated and continued to express the NSC marker Nestin (1.60±0.61 in AEA-treated cells vs 11.83±7.04 in control cells) (Fig. 2A). On the other hand, no statistically significant changes in the oligodendrocyte population were observed (Fig. 2A). These results suggest that *in vitro* AEA enhances differentiation of NSCs toward the neuronal lineage.

AEA can bind to both CB_1_ and CB_2_ receptors [9], [15]. Since we showed that both receptors are expressed in murine NSCs, albeit at different levels, we used selective antagonists and agonists for each receptor in order to highlight their differential contribution. Treatment of NSCs with AEA together with the CB_1_ receptor antagonist AM251 (1 µM) significantly reduced the percentage of differentiated neurons (16.20±1.23 with AEA+AM251 vs 21.79±1.12 with AEA, Fig. 2B). By contrast, treatment with the CB_2_ receptor antagonist AM630 (1 µM) did not significantly affect AEA-induced neuronal differentiation (Fig. 2B). Next, we treated NSCs with specific agonists of these receptors. In line with the results obtained with the selective antagonists, we found that the CB_1_ receptor agonist ACEA (1 µM) induced a statistically significant increase in differentiated neurons compared to control NSCs (17.31±2.20 with ACEA vs 9.93±2.72 in the control condition), which was abolished by co-treatment with the CB_1_ receptor antagonist AM251 (Fig. 2B). By contrast, the selective CB_2_ receptor agonist JWH133 (1 µM) was ineffective under the same conditions (Fig. 2B). These results suggest that activation of the CB_1_ receptor promotes neuronal differentiation of murine NSCs.

### ACEA enhances differentiation of neurons in culture

**Figure 3:**
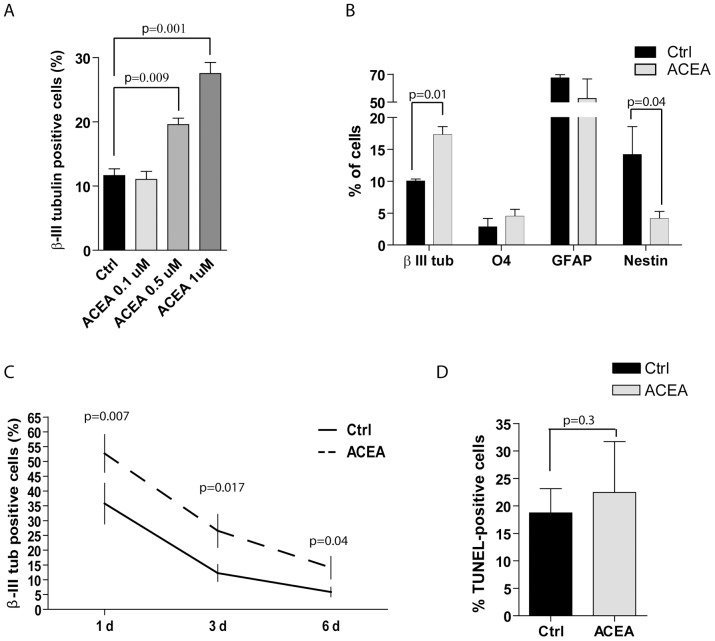
Selective activation of CB_1_ receptor is sufficient to promote neuronal differentiation of NSCs. (**A**) Dose response of the effect of ACEA (0.1–1 µM) on the neuronal differentiation of NSCs *in vitro*. (**B**) Quantitative analysis of the effect of 1 µM ACEA on the differentiation of NSCs in neurons (β-III tubulin-positive cells), oligodendrocytes (O4-positive cells), astrocytes (GFAP-positive cells) and progenitor cells (Nestin-positive cells) in control (black bars) and AEA-treated (grey bars) cells. (**C**) Time course analysis (1–6 days) of the effect of ACEA on the neuronal differentiation of NSCs (expressed as % of β-III tubulin-positive cells). (**D**) Quantitative analysis of the effect of ACEA on apoptosis of NSCs undergoing differentiation. Apoptosis was measured by immunofluorescence analysis of DNA fragmentation (TUNEL assay) in control (black bars) and AEA-treated (grey bars) cells. Data of all the experiments represent the mean ± SD of at least 3 independent experiments. Statistical analysis was performed using the paired t-test; the p value is indicated above the individual samples and the bars grouped by brackets.

To further explore the role of CB_1_ receptor in NSC differentiation, we studied the effect of its selective and potent agonist ACEA in more detail. As previously observed for AEA, treatment of NSCs with ACEA under proliferation conditions exerted a small but significant increase in the percentage of β-III tubulin-positive cells (3.43±0.91 with ACEA vs 1.25±0.32 in control cells) (Figure S1B). Furthermore, when NSCs were cultured under differentiation conditions [30], ACEA also augmented the percentage of neurons in a dose-dependent manner (Fig. 3A), whereas it did not significantly affect differentiation of NSCs toward astrocytes or oligodendrocytes (Fig. 3B). Importantly, like for the treatment with AEA, this CB_1_ receptor agonist strongly decreased the percentage of Nestin-positive precursor cells (Fig. 3B), further suggesting that it promotes their differentiation into the neuronal lineage.

Analysis of NSC differentiation was performed three days after plating the cells in differentiation medium. As under these conditions NSC proliferation is minimal if not absent, an increase in the percentage of neurons at this stage may derive from either increased differentiation of progenitor cells or increased survival of cells that have entered the neuronal differentiation pathway. To better understand the dynamics of the ACEA-induced effect on NSC differentiation, we performed a time-course analysis. When cells were stained at day 1, 3 and 6 after the onset of differentiation, ACEA significantly increased the percentage of β-III tubulin-positive cells already at day 1 (Fig. 3C), when positive cells have not yet acquired the morphology of neuronal cells (Figure S2A). The β-III tubulin-positive cells steadily declined from day 1 to day 6 in both control and ACEA-treated cells, but the ACEA-treated culture significantly contained an higher percentage of neurons for the whole duration of the experiment. Moreover, TUNEL analysis of apoptotic cells in the whole population at day 3 did not reveal significant differences in the two samples (Fig. 3D). These results suggest that CB_1_ receptor activation directly promotes differentiation of NSCs into neurons rather than enhancing their survival in culture.

### ACEA promotes maturation of neurons in culture

**Figure 4:**
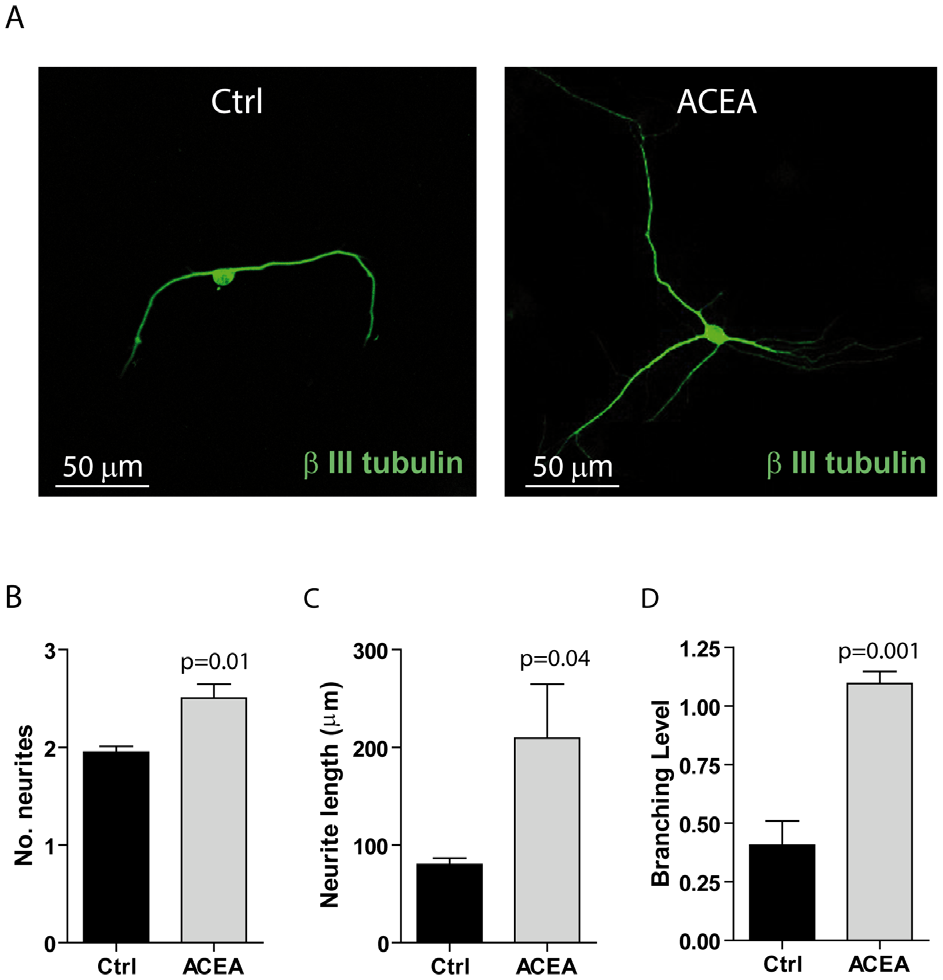
CB_1_ receptor stimulation enhances maturation of the *in vitro* differentiated neurons. (**A**) Representative images of the immunofluorescence analysis of β-III tubulin in cells differentiated from NSCs after three days of culture in 1% FBS in the presence or absence of 1 µM ACEA. (**B–D**) Bar graphs represent quantitative data of morphometric analyses of number (**B**), length (**C**) and branching level of neurites (**D**) in cells treated as described in (**A**). Data represent the mean ± SD of 4 experiments. Statistical analysis was performed using the paired t-test; the p value is indicated above the individual samples.

During the early stages of *in vitro* development, neurons initially grow processes across the surface matrix and then develop the axon and the dendrites, called together neurites. These protrusions increase in length and complexity, thus displaying numerous secondary and tertiary branching [33], [34] (Figure S3A). In order to evaluate the features of neuron protrusions, neurite number, length and complexity was evaluated in detail by morphological and morphometric examination (see Materials and Methods and Figure S3B,C). Primary neurites were defined as processes projecting directly from the cell body (branching level 0), secondary neurites were processes that branched from any primary neurite (branching level 1) and tertiary processes were those that projected from any secondary neurite (branching level 2) (Figure S3B,C). We observed that the neurons obtained in the presence of ACEA displayed a higher degree of maturation (Fig. 4A), as indicated by the increased number (2.51±0.25 vs 1.95±0.14 in control; Fig. 4B) and length (209.51±95.90 µm vs 80.10±11.14 µm in control; Fig. 4C) of the neurites. Moreover, ACEA also enhanced neuron complexity, as indicated by the higher branching level (1.00±0.08 vs 0.42±0.17 in control; Fig. 4D). These results indicate that activation of CB_1_ during NSC differentiation *in vitro* promotes the maturation of neurons.

### Intracellular signalling involved in ACEA-induced NSC differentiation

**Figure 5:**
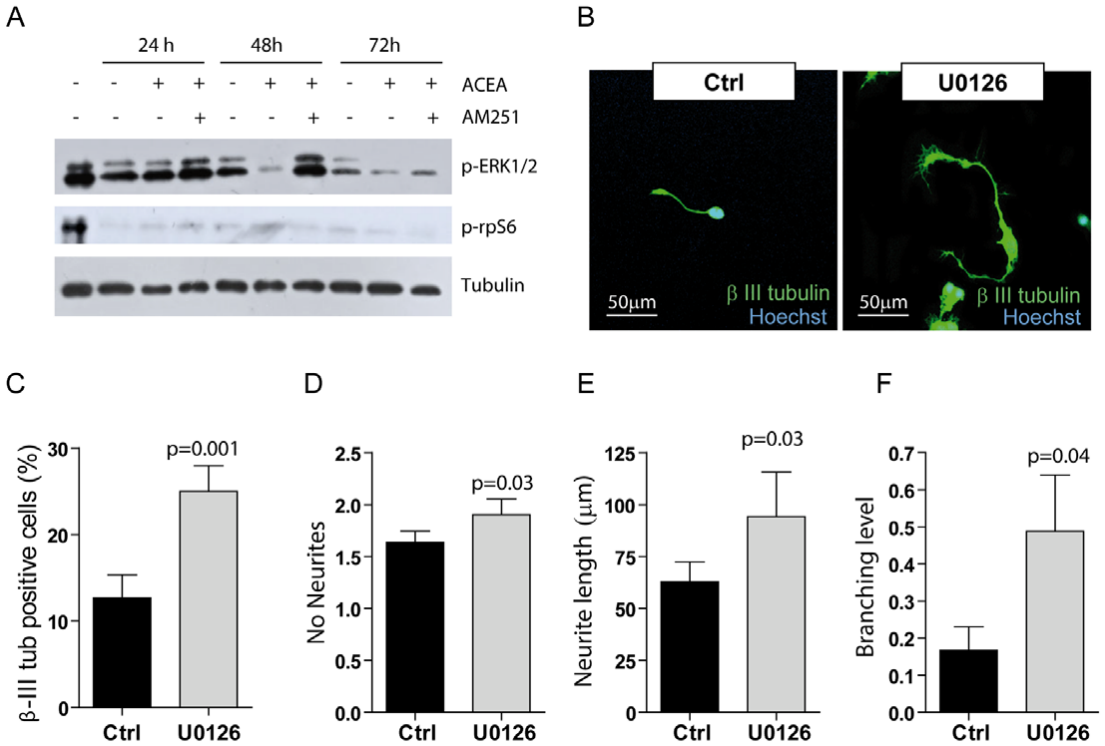
Reduced activity of ERK1/2 kinases promotes neuronal differentiation of NSCs. (**A**) Western blot analysis of the phosphorylation levels of ERK1/2 (p-ERK1/2) and rpS6 (p-rpS6), a downstream target of the mTOR pathway. Cell extracts (30 µg) from NSCs treated with or without ACEA (1 µM) and the CB_1_ antagonist AM251 (1 µM) in the presence of 1% FBS for the indicated time were analysed. (**B**) Representative images of the immunofluorescence analysis of β-III tubulin in cells differentiated from NSCs after three days of culture in 1% FBS in the presence or absence of 10 µM U0126, a selective inhibitor of the ERK1/2 pathway. (**C**) The bar graph represents quantitative data (expressed in % of β-III tubulin-positive cells) of the immunofluorescence analysis of neuronal differentiation of NSCs cultured in differentiating condition in the presence or absence of 10 µM U0126. (**D–F**) Bar graphs represent quantitative data of morphometric analyses of the number (**D**), the length (**E**), and branching level of neurites (**F**) in cells treated as described in (**C**). Data represent the mean ± SD of 6 experiments. Statistical analysis was performed using the paired t-test; the p value is indicated above the individual samples.

Next, we sought to investigate the signal transduction pathway(s) involved in CB_1_ receptor-induced NSC differentiation. One of the signal transduction pathways triggered by G-proteins coupled to CB_1_ and CB_2_ receptors in other cell systems is the mitogen activated protein kinase (MAPK) cascade that impinges on phosphorylation and activation of ERK1 and 2 (ERK1/2) [35]. Another signalling route activated by the CB_1_ receptor in hippocampal neurons is the mTOR/p70S6K pathway, which mediates the modulatory effects of eCBs on long-term memory [17]. Thus, we investigated the phosphorylation levels of ERK1/2 and of the ribosomal protein S6 (rpS6), the main downstream substrate of the mTOR/p70S6K pathway, during NSCs differentiation. Western blot analysis of protein extracts obtained after 1, 2 and 3 days of culture showed that the levels of ERK1/2 phosphorylation decline during NSC differentiation (Fig. 5A). By contrast, phosphorylation of rpS6 is detected in undifferentiated NSCs (T0 in Fig. 5A) but absent during the whole time-course of differentiation *in vitro*, suggesting that the mTOR pathway is turned off early during this process. Interestingly, ACEA caused a further reduction in ERK1/2 phosphorylation at 48 and 72 hours of culture (Fig. 5A), and this effect was blocked (48 hours) or partially reverted (72 hours) by the CB_1_ receptor antagonist AM251 (Fig. 5A). Neither ACEA nor AM251 affected the phosphorylation of rpS6 (Fig. 5A). Other kinases involved in cell growth and differentiation, such as the p38 MAPK and AKT, were either not phosphorylated during NSC differentiation (p38) or affected by ACEA only transiently (AKT at 48 hours) (Figure S3D). Notably, we observed an early response activation of ERK1/2 after 15 minutes of treatment with ACEA, while no phosphorylation of AKT was observed (Figure S3E). Thus, inhibition of the ERK1/2 pathway during NSC differentiation may results from enhanced desensitization of the pathway by prolonged exposure to ACEA. Phosphorylation of ERK1/2 by various upstream signal transduction pathways causes their activation [36]. To directly correlate neural differentiation with the observed decrease in ERK1/2 activity, NSCs were treated with U0126 (10 µM), a selective inhibitor of the ERK1/2 pathway [37], during differentiation. Immunofluorescence analysis of the β-III tubulin positive cells (Fig. 5B) showed that inhibition of the ERK1/2 pathway promotes neuronal differentiation (Fig. 5C). Furthermore, morphological and morphometric examination indicated that treatment with U0126 also increased neuronal maturation, as monitored by the augmented number (1.90±0.36 with U0126 vs 1.62±0.20 in control cells; Fig. 5D), length (94.20±52.21 µm with U0126 vs 62.91±23.00 µm in control cells, Fig. 5E) and complexity of neurites (branching level 0.48±0.30 with U0126 vs 0.16±0.11 in control cells; Fig. 5F). These results indicate that reduced ERK1/2 activity promotes neuronal differentiation and maturation and suggest that CB_1_ exerts its effects through inhibition of this pathway in mouse NSCs.

### Activation of the CB_1_ receptor modulates the expression of select genes during neuronal differentiation of NSCs

**Figure 6:**
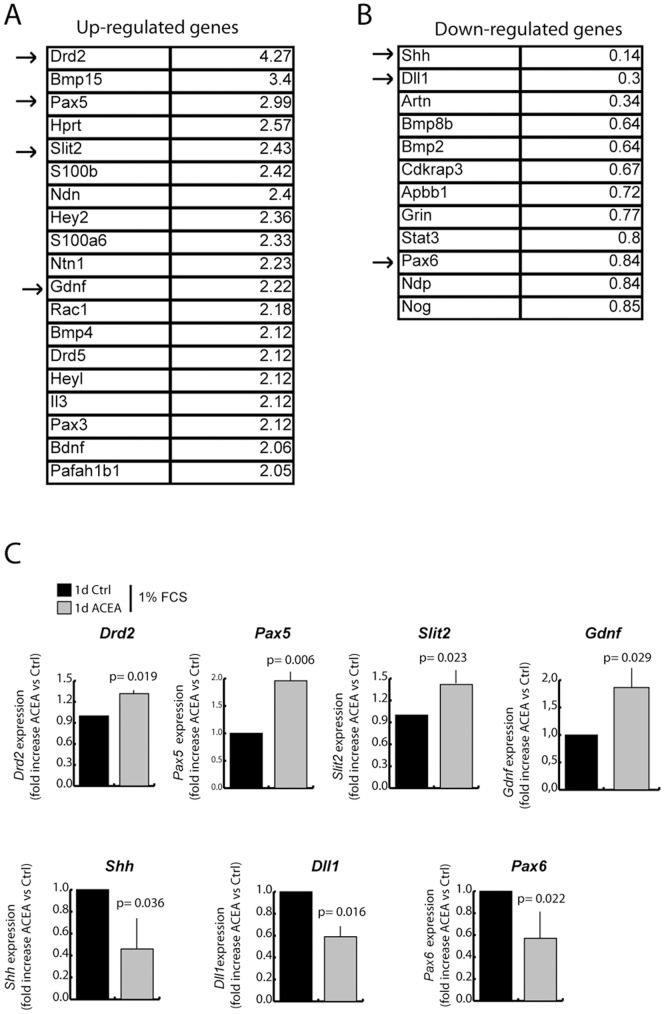
CB_1_ receptor activation during neuronal differentiation of NSCs modulates gene expression. (**A–B**) Results of the Neurogenesis and Neural Stem Cell PCR array. List of the genes up-regulated (**A**) and down-regulated (**B**) in NSCs undergoing differentiation for 24 hours in the presence of 1% FBS and 1 µM ACEA, obtained from the PCR Array analysis. (**C**) Validation of the results of the PCR array by qPCR analysis of seven genes (*Drd2*, *Pax5*, *Slit2*, *Gdnf*, *Shh*, *Dll1* and *Pax6*) selected for their relevance for neuronal differentiation or NSC stemness. Data (mean ± SD of 5 experiments) are expressed as fold increase of ACEA-treated versus control samples, using *Gapdh* expression as standard control (see Materials and Methods). Statistical analysis was performed using the paired t-test; the p value is indicated above the individual samples.

Neuronal differentiation is guided by extensive changes in gene expression [38], [39]. To test whether treatment with ACEA affected gene expression in NSCs undergoing differentiation, we profiled the expression levels of 84 genes known to be involved in neurogenesis and NSC biology (see Materials and Methods). We performed qPCR analysis of RNA extracted from NSCs cultured under differentiation conditions for 24 hours in the presence or absence of ACEA. Using an arbitrary cut-off of two-fold change, expression of 19 genes in the array resulted up-regulated by activation of CB_1_ during NSC differentiation (Fig. 6A). By contrast, few genes were downregulated by CB_1_ receptor activation. Using a less stringent cut-off (0.85 fold change), we identified 12 genes that were down-regulated when differentiation was carried out in the presence of the CB_1_ receptor agonist (Fig. 6B). To validate the results of the PCR array, we re-analyzed 7 selected genes (4 upregulated and 3 downregulated by treatment with ACEA) by qPCR using different sets of primers (Fig. 6C; Table 1). Noteworthy, ACEA modulated the expression of genes involved in neural cell fate determination and in neurite outgrowth (Table 2). For instance, ACEA induced expression of *Pax5*, which is expressed in the developing midbrain where dopaminergic neurons are generated [40], of *Slit2*, a protein that promotes axonal projections [41], [42], of *Drd2*, which encodes the dopamine receptor D2 [43], and of *Gdnf*, a glial derived neurotrophic factor that promotes differentiation of the neurons [44]. On the other hand, ACEA reduced the expression of factors that regulate neural progenitor cell proliferation (Table 2), such as the signalling molecule Sonic Hedgehog, encoded by *Shh* [45], *Pax6* [46] and the NOTCH ligand Delta like 1, encoded by *Dll1* [47]. Moreover, we found that ACEA repressed the expression of the NSC markers *Nestin* [48] and *Sox2* [49], which were not present in the PCR array (Figure S3F). These results suggest that activation of CB_1_ during NSC differentiation promotes the expression of genes involved in the commitment toward neuronal lineage and in the maturation of neurons, while repressing genes required for NSC proliferation and self-renewal.

**Table 2:** Description of selected genes modulated by ACEA.

Gene	Description	Role in neurogenesis
*Upregulated genes*		
***Pax5*** (NM_008782.2)	Paired box gene 5; transcription factor	Pax5 positive cells are source of midbrain dopaminergic neurons [41].
***Slit2*** (NM_178804.3)	Slit homologue 2; Secreted protein	Role in axon guidance and neuronal migration [42], [43].
***Drd2*** (NM_010077.2)	Dopamine receptor D2; G protein-coupled receptor	Marker of dopaminergic neurons [44].
*Downregulated genes*		
***Shh*** (NM_009170.3)	Sonic hedgehog; secreted peptide	Involved in neural stem cell self-renewal [46].
***Pax6*** (NM_001244200.1)	Paired box gene 6; Transcription factor	Regulation of neurogenesis [47].
***Dll1*** (NM_007865.3)	Delta-like 1; Transmembrane protein; Notch ligand	Increased during neuronal differentiation [48].
***Nestin*** (NM_016701.3)	Nestin; intermediate filament protein	Neural progenitor marker [49].
***Sox2*** (NM_011443.3)	SRY-box containing gene 2; Transcription factor	Neural stem cell marker [50].

## Discussion

Neurogenesis is modulated by different factors produced by the surrounding environment. An important modulatory action in the central nervous system is played by eCBs. In the present study we describe the pro-differentiation effect exerted by activation of CB_1_ receptor in NSCs. In particular, CB_1_ agonists favored differentiation of neural progenitors into neurons and enhanced their maturation in culture. This effect was accompanied by reduced activity of the ERK1/2 pathway and by changes in gene expression that support neuronal differentiation and morphological maturation. Thus, our findings uncover a potential role of CB_1_ receptor in the modulation of mouse NSC fate.

Previous studies have mainly focused their attention of the effect played by the CB_1_ receptor in the regulation of NSC proliferation. First, it was shown that AEA promotes proliferation of neural progenitors through activation of CB_1_ [23]. Moreover, activation of CB_1_ stimulated embryonic and adult hippocampal neurogenesis *in vivo* (22), whereas CB_1_ knockout mice displayed decreased hippocampal neurogenesis [24]. In addition to stimulating NSC proliferation, a recent report suggested that both CB_1_ and CB_2_ receptors promote NSC migration from the SVZ in the postnatal brain [50]. This finding is particularly relevant in light of a possible role for eCBs in supporting migration of newly formed neurons to areas of brain injury. On the other hand, the contribution of eCBs to NSC differentiation is much less clear. Treatment with AEA of neural progenitors isolated from postnatal rats caused an increase in the number of GFAP-positive cells [51], whereas a small increase in β-III tubulin-positive cells was observed in post-natal mouse neural progenitors treated with AEA [26]. Thus, given these apparently discordant results, we have set out to investigate this issue in further detail.

We have isolated a self-renewing population from the cortex of E13.5 mouse embryos. These cells display features of NSCs [32], as they maintain clonogenic potential and differentiate into neurons, astrocytes and oligodendrocytes under proper conditions. We found that these cells express both CB_1_ and CB_2_ receptors, with the former being the most abundant. Treatment of NSCs with AEA under conditions that favour their differentiation increased the percentage of β-III tubulin-positive neurons by about 2-fold, whereas a small decrease in GFAP-positive astrocytes was observed. Remarkably, AEA also strongly decreased the percentage of cells retaining stemness features (Nestin-positive cells), suggesting that it accelerates their commitment to differentiate into neurons. Although this effect was also observed when NSCs were cultured under proliferating conditions, the percentage of cells acquiring the neuronal marker was limited, suggesting that AEA is not sufficient to commit NSCs into the neuronal lineage, but it rather enhances their differentiation under proper conditions. By using receptor-selective agonists and antagonists, we showed that CB_1_ receptor participates to the AEA-induced effect, whereas stimulation or inhibition of the CB_2_ receptor did not affect NSC neuronal differentiation under our experimental conditions. However, it is worth recalling that AEA is also known to bind and activate other non-CB_1_ non-CB_2_ receptors, such as the G protein-coupled receptor GPR55, also known as CB_3_ [52], the ionotropic transient receptor potential vanilloid channels [53], and the nuclear peroxisome proliferator-activated receptors [54]. Thus, a possible contribution of these additional receptors to the effects of AEA reported here cannot be ruled out. Nevertheless, in this study we sought to focus on the direct contribution of the two major cannabinoid receptors to NSC differentiation.

We found that ACEA, a selective CB_1_ agonist, could profoundly influence neuronal maturation (neurite length and complexity). Notably, a similar increase in neurite complexity was also reported for migrating neurons exposed to ACEA [50]. Neurite outgrowth is required for neuronal connectivity and disruption of this process can cause human cognitive deficits, such as Huntington's chorea and Alzheimer's disease [55]. Thus, due to its primary importance for the development and the functionality of the nervous system, neurite outgrowth has been used as an important parameter to assess neuronal differentiation [34]. Our findings that CB_1_ activation strongly promotes neuronal maturation and complexity might suggest that eCBs participate to the formation of new neuronal circuits in the developing brain or as a consequence of brain injury. In line with this hypothesis, it was shown that ablation of CB_1_ gene caused impaired neurogenesis *in vivo* [24], whereas chronic treatment with synthetic cannabinoids elicited anxiolytic- and anti-depressant effects through the promotion of *de novo* neurogenesis in the hippocampus [22].

Neuronal differentiation is orchestrated by extensive changes in gene expression [38], [39]. To gain insights into the mechanisms triggered by eCBs in NSCs, we profiled the expression of 84 genes with relevance for NSC biology and/or neurogenesis during differentiation in the presence of ACEA. Remarkably, we found that activation of CB_1_ caused the upregulation of several genes involved in neuronal differentiation. For instance, *Pax5* encodes a transcription factor expressed in the region and at the developmental stage when dopaminergic neurons appear in the brain [40], and ACEA increased *Pax5* expression together with that of *Gdnf*, a glial-derived growth factor that drives differentiation of neural progenitors into neurons [44], and of *Drd2*, which encodes the dopamine receptor D2 [43]. These results suggest that activation of CB_1_ may direct *in vitro* differentiation of NSCs toward neurons by selective upregulation of a subset of genes involved in this process. Notably, the upregulation of *Gdnf* suggests that the CB_1_ receptor may also be expressed by the glial cells present in the population undergoing differentiation. In turn, *Gdnf* activation in glial cells may direct them to promote differentiation and maturation of the surrounding neurons, raising a potential interest for biomedical applications. Another interesting gene upregulated by ACEA is *Slit2*, which encodes a repulsive ligand for the ROBO receptor. *Slit2* promotes axonal projections in developing brain [41], [42], suggesting that it may take part to the increase in neurite outgrowth observed upon CB_1_ activation *in vitro*. On the other hand, ACEA reduced the expression of intrinsic (*i.e.* the transcription factor SOX2) and extrinsic factors (*i.e.* the signalling molecule SHH) involved in self-renewal of NSCs [45], or of genes that are downregulated when neuronal precursors are engaged in differentiation, such as *Pax6* [46]. Thus, these observations further support the hypothesis that activation of CB_1_ promotes the commitment of NSCs toward neuronal lineage and enhance the maturation of the developing neurons.

Differentiation of NSCs *in vitro* was accompanied by a reduction in the phosphorylation status of ERK1/2 kinases after prolonged incubation (48 and 72 hours) and treatment with ACEA further decreased ERK1/2 phosphorylation at these time points. We also observed a transient increase in AKT phosphorylation after 48 hours of treatment with ACEA. Since the AKT pathway was shown to control dendritic branching in rat hippocampal neurons [56], it is possible that the transient activation of this pathway elicited by ACEA contributes to the enhanced differentiation and maturation of NSCs. On the other hand, downregulation of the ERK1/2 activity appears to be directly involved in the positive action of CB_1_ activation on neuronal differentiation, because we observed that pharmacological inhibition of this pathway could recapitulate the effects elicited by ACEA. Importantly, a recent study linked the temporally regulated inhibition of the FGF/ERK pathway with neural differentiation of stem cells [57]. In particular, the temporal modulation of the FGF/ERK pathway was crucial for the efficient production of functional neurons [57]. This might be true also for CB_1_ activation in NSCs. Indeed, as also documented by other studies [35], we found that ACEA transiently increased phosphorylation of ERK1/2 in the first hour of stimulation (Figure S2D), whereas inhibition of the pathway was observed after prolonged incubation with the drug. Thus, it is possible that the dual effect of CB_1_ activation on ERK1/2 phosphorylation also underlies its role in neuronal differentiation of NSCs. Although the exact signal transduction events triggered downstream of CB_1_ activation have not been elucidated in our study, these observations suggest that the observed upregulation of genes involved in differentiation of neurons might be driven by the decrease in ERK1/2 phosphorylation caused by treatment with ACEA. Future studies will be directed at specifically addressing this issue.

## Conclusions

In conclusion, our study highlights a novel contribution of CB_1_ receptor to the regulation of NSC neuronal differentiation and maturation *in vitro*. In addition to the previously reported effects on NSC proliferation and migration [22], [50], our work suggests that eCBs may also support repair of brain injuries through the enhanced differentiation of precursor cells into mature neurons.

## Supporting Information

Figure S1 **Effect of AEA and ACEA on neuronal differentiation of NSCs.** NSCs cultured under proliferation conditions were treated with or without anandamide (AEA 1 µM) or ACEA (1 µM) for three days. (A) The bar graph represents quantitative data (expressed in % of cells) of the immunofluorescence analysis of the effect exerted by AEA on NSCs. Immunofluorescence analysis was performed as described in Figure 1C to detect neurons (β-III positive cells), oligodendrocytes (O4-positive cells), astrocytes (GFAP-positive cells) and progenitor cells (Nestin-positive cells) in control (black bars) and AEA-treated (grey bars) cells. Data represents the mean ± standard deviation (SD) of 3 experiments. Statistical analysis was performed using the paired t-test; p value is reported above the bars. The percentage of cells positive for the neuronal differentiation marker β-III tubulin (differentiating neurons) were significantly increased in AEA treated NSCs (4.76±0.90 vs 2.26±0.50). (B) The bar graph represents quantitative data (expressed in % of cells) of the immunofluorescence analysis of the effect exerted by ACEA on NSCs. Data are reported as described in (A). ACEA significantly increases the percentage of β-III tubulin positive cells (3.43±0.91 vs 1.25±0.32) (n = 3).
(TIF)

Figure S2 **Time-course analysis of ACEA-induced differentiation of NSCs.** Representative images of β-III tubulin cells from NSCs after one, three or six days of culture in 1% FBS in the presence or in the absence of 1 µM ACEA.
(TIF)

Figure S3 **ACEA promotes neuronal differentiation and maturation.** (A) Representative images of β-III tubulin-positive neurons differentiated from NSCs after three days in the absence (Ctrl) or in the presence of ACEA (ACEA). (B) Schematic representation of three neurons displaying different branching levels, according to the presence of primary (branching level 0), secondary (branching level 1) or tertiary neurites (branching level 2). (C) Representative images of β-III tubulin-positive neurons obtained from control or ACEA-treated NSC cultures. Their corresponding branching level is indicated in the inset in the top right corner. (D,E) Western blot analyses of the phosphorylation levels of AKT (p-AKT) and p38 (p-p38) (D) or of ERK1/2 (p-ERK1/2) (E) in cell extracts (30 µg) from NSCs treated with or without ACEA (1 µM) in the presence of 1% FBS for the indicated time. (F) Real time PCR analysis of Nestin and Sox2 gene expression in NSCs cultured for 24 hours with or without ACEA (1 µM).
(TIF)
