# Supplementary figures and images for: Type-1 (CB1) Cannabinoid Receptor Promotes Neuronal Differentiation and Maturation of Neural Stem Cells
Source: PLoS One. 2013 Jan 23;8(1):e54271. doi: 10.1371/journal.pone.0054271 (PMC3553153; doi:10.1371/journal.pone.0054271)

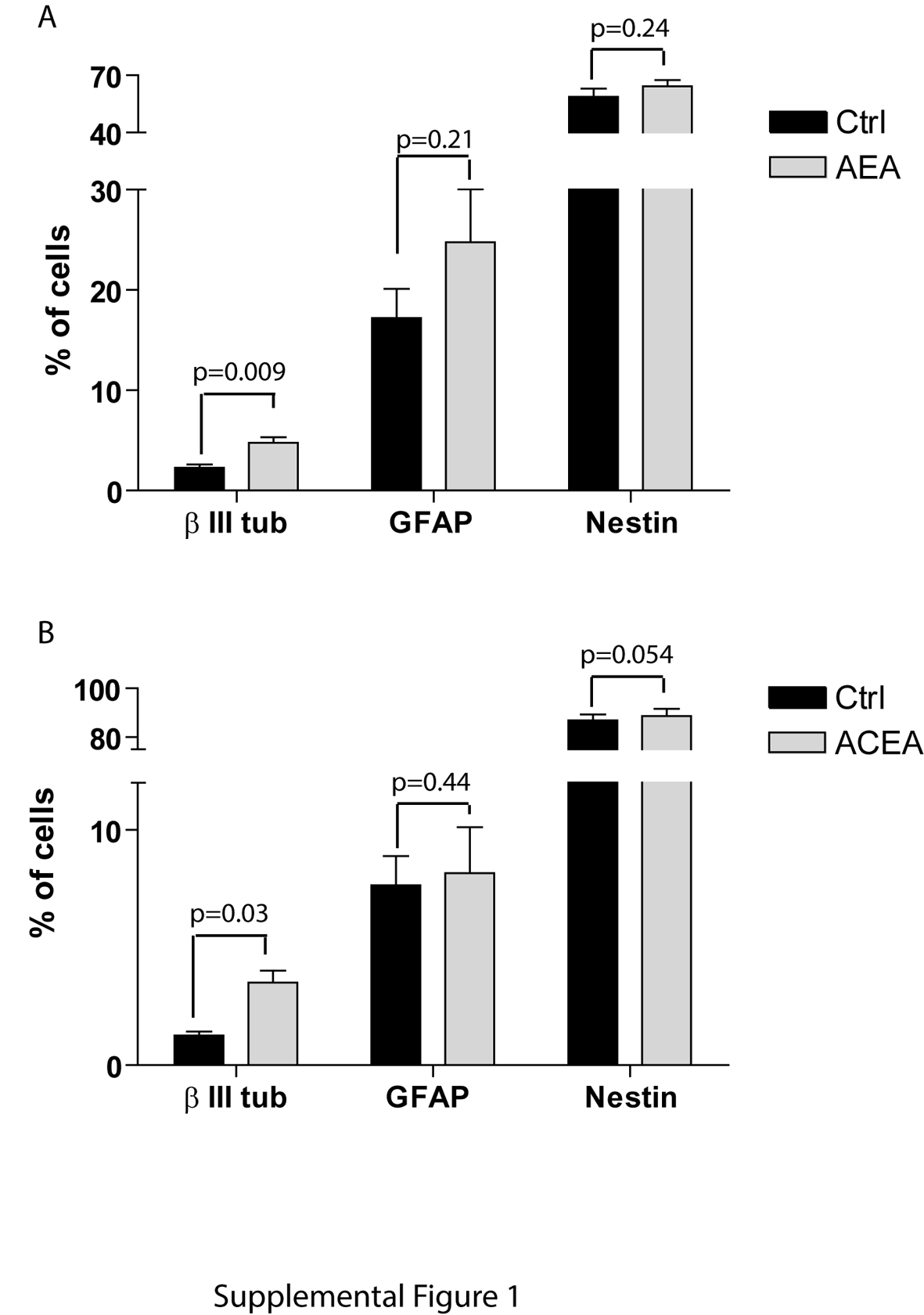

Supplement: Figure S1 — Effect of AEA and ACEA on neuronal differentiation of NSCs. NSCs cultured under proliferation conditions were treated with or without anandamide (AEA 1 µM) or ACEA (1 µM) for three days. (A) The bar graph represents quantitative data (expressed in % of cells) of the immunofluorescence analysis of the effect exerted by AEA on NSCs. Immunofluorescence analysis was performed as described in Figure 1C to detect neurons (β-III positive cells), oligodendrocytes (O4-positive cells), astrocytes (GFAP-positive cells) and progenitor cells (Nestin-positive cells) in control (black bars) and AEA-treated (grey bars) cells. Data represents the mean ± standard deviation (SD) of 3 experiments. Statistical analysis was performed using the paired t-test; p value is reported above the bars. The percentage of cells positive for the neuronal differentiation marker β-III tubulin (differentiating neurons) were significantly increased in AEA treated NSCs (4.76±0.90 vs 2.26±0.50). (B) The bar graph represents quantitative data (expressed in % of cells) of the immunofluorescence analysis of the effect exerted by ACEA on NSCs. Data are reported as described in (A). ACEA significantly increases the percentage of β-III tubulin positive cells (3.43±0.91 vs 1.25±0.32) (n = 3). (TIF) [file pone.0054271.s001.tif]

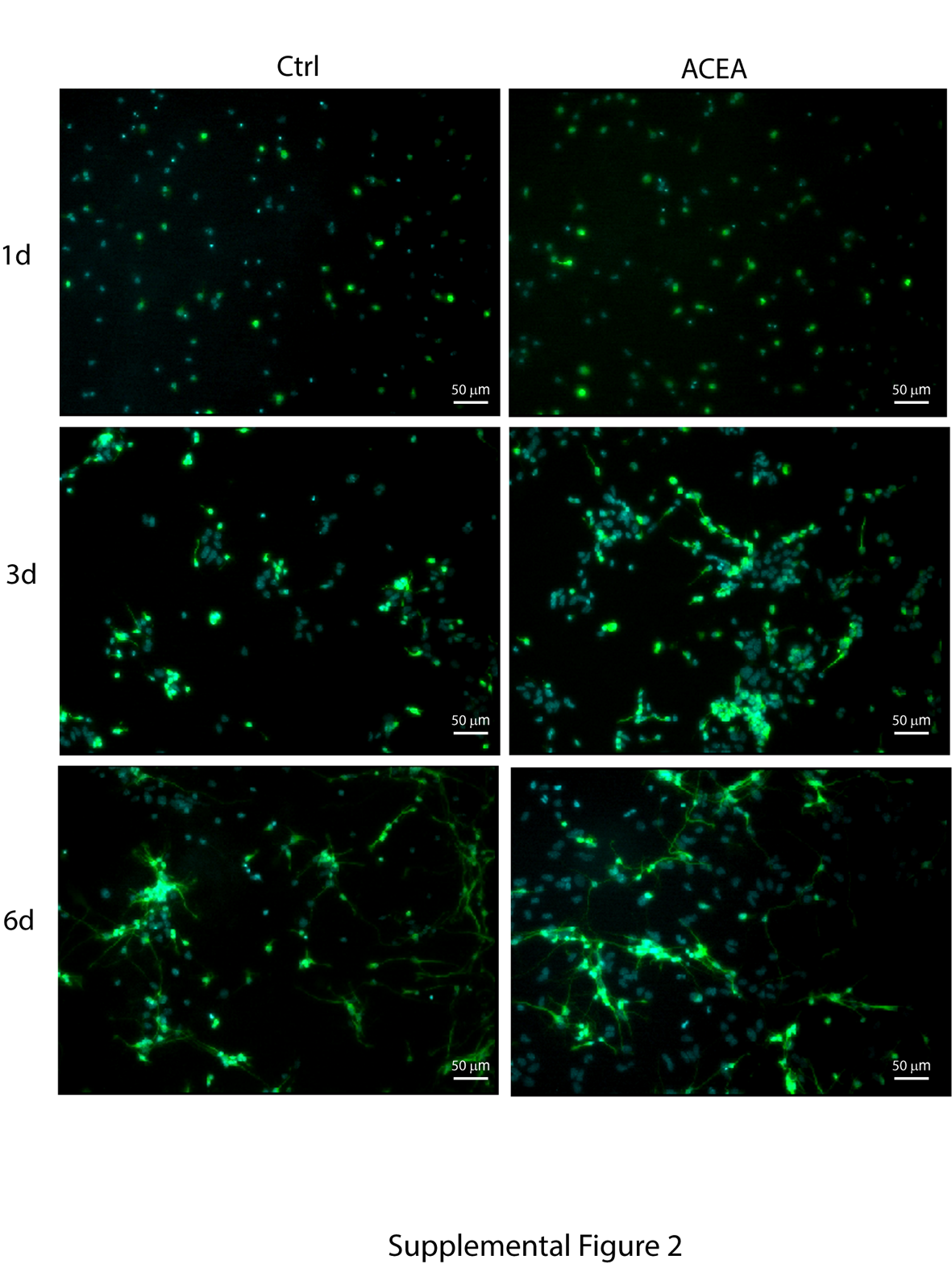

Supplement: Figure S2 — Time-course analysis of ACEA-induced differentiation of NSCs. Representative images of β-III tubulin cells from NSCs after one, three or six days of culture in 1% FBS in the presence or in the absence of 1 µM ACEA. (TIF) [file pone.0054271.s002.tif]

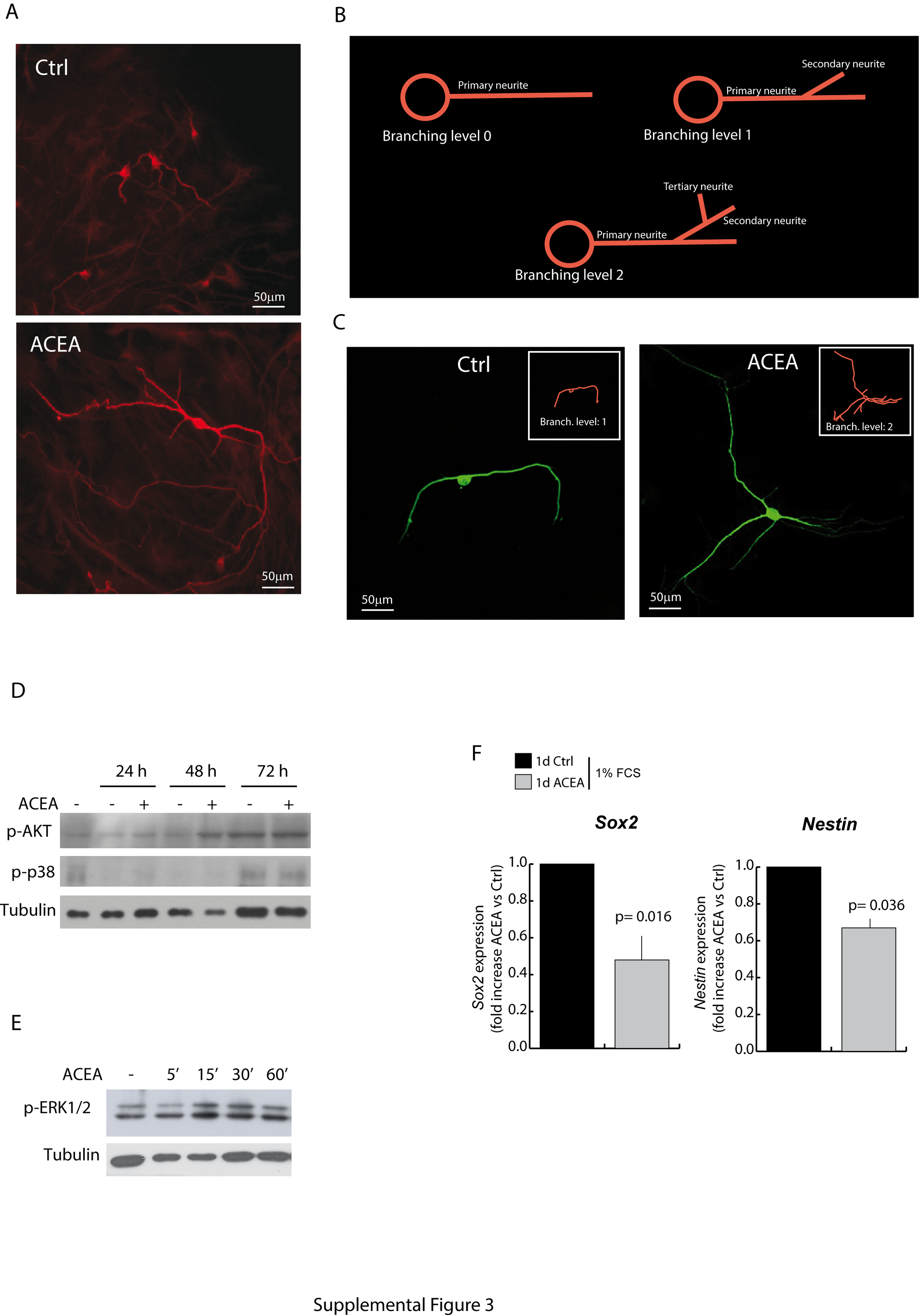

Supplement: Figure S3 — ACEA promotes neuronal differentiation and maturation. (A) Representative images of β-III tubulin-positive neurons differentiated from NSCs after three days in the absence (Ctrl) or in the presence of ACEA (ACEA). (B) Schematic representation of three neurons displaying different branching levels, according to the presence of primary (branching level 0), secondary (branching level 1) or tertiary neurites (branching level 2). (C) Representative images of β-III tubulin-positive neurons obtained from control or ACEA-treated NSC cultures. Their corresponding branching level is indicated in the inset in the top right corner. (D,E) Western blot analyses of the phosphorylation levels of AKT (p-AKT) and p38 (p-p38) (D) or of ERK1/2 (p-ERK1/2) (E) in cell extracts (30 µg) from NSCs treated with or without ACEA (1 µM) in the presence of 1% FBS for the indicated time. (F) Real time PCR analysis of Nestin and Sox2 gene expression in NSCs cultured for 24 hours with or without ACEA (1 µM). (TIF) [file pone.0054271.s003.tif]
